# ‘Research clinics’: online journal clubs between south and north for student mentoring

**DOI:** 10.3402/gha.v9.30434

**Published:** 2016-10-06

**Authors:** Salla Atkins, Dinansha Varshney, Elnta Meragia, Merrick Zwarenstein, Vishal Diwan, for the ARCADE consortium

**Affiliations:** 1 Department of Public Health Sciences, Karolinska Institutet, Stockholm, Sweden; 2 Department of Public Health and Environment, R.D. Gardi Medical College, Ujjain, India; 3 Department of Family Medicine, Centre for Studies in Family Medicine, Schulich School of Medicine & Dentistry, Western University, London, ON, Canada; 4 International Centre for Health Research, Ujjain Charitable Trust Hospital and Research Centre, Ujjain, India

**Keywords:** capacity building, postgraduate education, global health, developing countries

## Abstract

**Background:**

Capacity development in health research is high on the agenda of many low- and middle-income countries.

**Objective:**

The ARCADE projects, funded by the EU, have been working in Africa and Asia since 2011 in order to build postgraduate students’ health research capacity. In this short communication, we describe one initiative in these projects, that of research clinics – online journal clubs connecting southern and northern students and experts.

**Design:**

We describe the implementation of these research clinics together with student and participant experiences.

**Results:**

From 2012 to 2015, a total of seven journal clubs were presented by students and junior researchers on topics related to global health. Sessions were connected through web conferencing, connecting experts and students from different countries.

**Conclusions:**

The research clinics succeeded in engaging young researchers across the globe and connecting them with global experts. The contacts and suggestions made were appreciated by students. This format has potential to contribute toward research capacity building in low- and middle-income countries.

## Introduction

Capacity development in health research is high on the agenda of many low- and middle-income countries (1). The 10/90 gap persists (2), and there is a global recognition that countries will not meet health goals if research capacity is not improved (3). The ARCADE projects (www.arcade-project.org), funded by the European Union, have been developing capacity in health systems and services research and in social determinants of research from 2011 to 2015, using various methods (4). The projects had 16 partners, which were research institutes and universities, across Africa, Asia, and Europe. The activities within the project centred on identifying institutional capacity in training young professionals to address health systems and social determinants of health research, developing and delivering courses to postgraduate students in partner institutes, and building capacity in grants management and communications at partner institutes (4).

The projects had a strong focus on mentoring students across institutions and across country borders, as part of mentoring doctoral students (5). However, the research community has noted that mentoring is not without challenges. Traditional workshops are resource and time intensive, and sufficient numbers of experts are not available in resource-constrained settings to mentor students (6). Many students are also active in their home health systems while studying, and may have difficulty in taking time off to attend workshops. Our mentoring process was intended to support a ‘pipeline’ of researchers at different stages of their careers, from masters training to postdoctoral work, through inter-researcher discussions, joint research groups, and programmes on research aimed at various problems and conditions.

One such initiative to support informal mentoring of young researchers was starting a series of ‘Research Clinic’ online seminars. These seminars were a platform of capacity building available globally, offering the opportunity for students to present their papers, protocols, or other in-progress work, to be given feedback by international experts. We wanted to bring together international leaders and researchers on health research topics with emerging, young leaders and researchers in low- and middle-income contexts, without straining either with international travel. Journal clubs have been held for more than a century, and to date, there is no decided format for holding one (7). Here, as organisers, mentors, and project coordinators, we report our experience of establishing and running one type of journal club, conducted online. In a process led by the first author, we examined our experience of seven such journal clubs, conducted over 3 years. We collected and reviewed reflections after each journal club from email and notes of face-to-face discussions. SA summarised these in the first version of this report, and these reflections were further added to by the other authors.

## The research clinics format

**Fig. 1:**
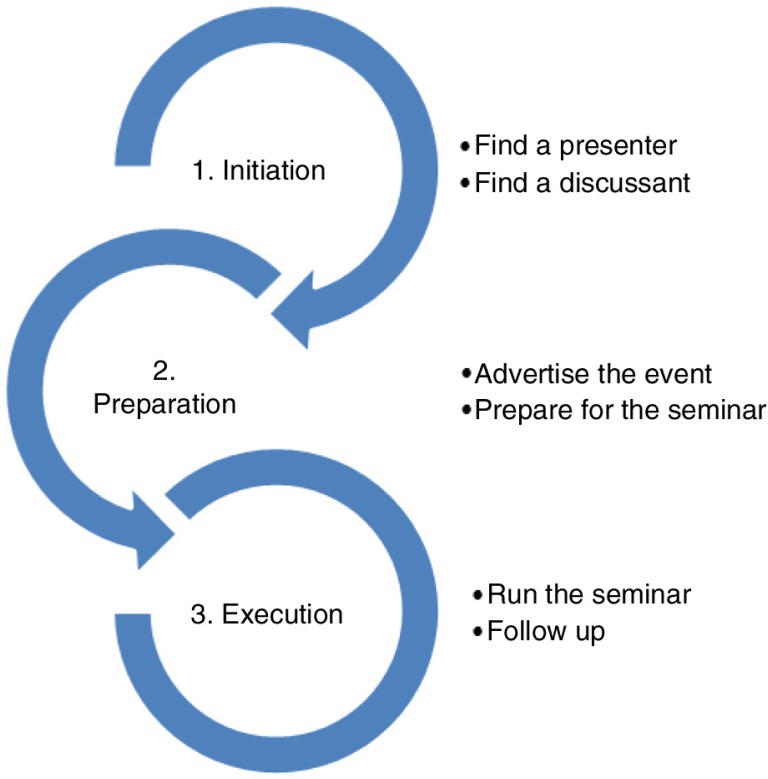
Steps in arranging a research clinic.

Arranging a research clinic required the following steps (Fig. 1).

**Fig. 2:**
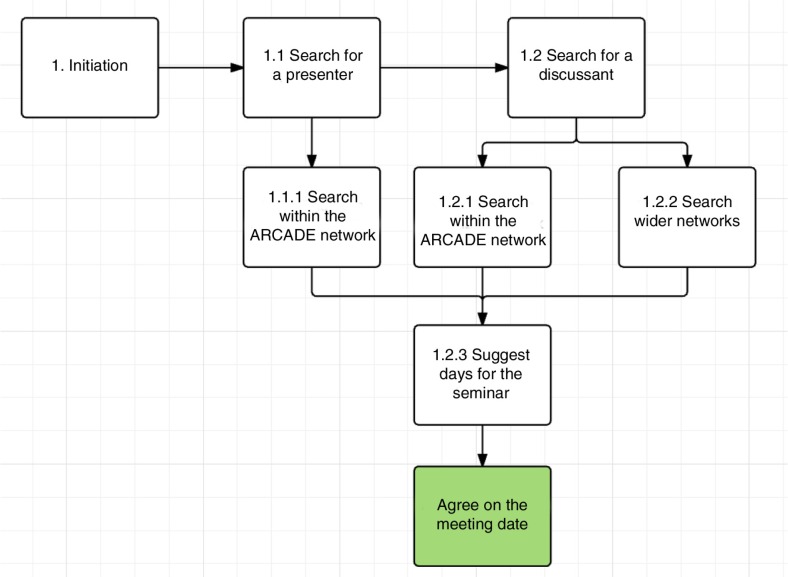
The research clinic arrangement process.

First, a presenter was identified from the ARCADE network. The presenter could be a student or young researcher at different stages of his/her career that could present his/her topic and would benefit from feedback on their topic. All students from the ARCADE projects’ partner institutions were eligible. After the presenter was found, an email was sent out to the project partners to identify a discussant, an expert in the area who can offer constructive feedback on the paper or presentation. In some settings, students were less forthcoming with proposals, concerned that the ideas could be stolen. The experts, on the other hand, were difficult to locate because of busy schedules, time differences, and lack of interest. Matching students’ topics and experts’ areas of interest was also a challenge. When both were identified, a date was agreed for the seminar (see Fig. 2). Most arrangements were done via email. Sending emails and follow-up reminders took approximately an hour; however, because most people replied when they could, the entire process of arranging a research clinic could be spread over a month.

Following the identification of the key people for the seminar, a number of steps needed to be taken at the appropriate times (approximately 1 month to 2 weeks before the seminar). First, proper advertising and promotion of the event was needed to ensure an audience. The invitation also contained guidelines on how to attend in order to ensure the seminar was smoothly run.

We used the following tools to advertise:

- social media, such as Facebook, ARCADE Twitter account, website, and LinkedIn group
- email
  - to ask PIs and project staff to share the event with their academic community
  - to ask that students representatives be invited to share the event with the rest of the students
- posters at universities
- personal communication

Concurrently with advertising a number of steps were carried out:

- The presenter prepared his/her presentation for the seminar
- The presenter sent the paper/work to the mentor at least 1 week before the seminar day
- The mentor read the paper/work and prepared discussion points

The seminars were run mainly through GoToMeeting software. All participants received a user guide to the software before the seminar. If necessary, a testing session was held a few days before the seminar in order to ensure that participants could use the software and had the necessary equipment.

Running the seminar was straightforward and rather similar to a within-institute journal club. The audience, presenter, and the mentor met online. The presenter presented the topic for a maximum of half an hour, the mentor discussed the paper and the presentation, and the audience commented on the study.

We conducted seven research clinics across the consortium from 2013 to 2015. As time passed, we experimented and used different methods to connect students, experts, and audiences. At the end of the process, instead of a single person using headphones and their computer to give a presentation or participate, whole rooms of researchers and students were connected using video cameras and computers. Each meeting lasted approximately 1 hour.

## The topics of research clinics

Table 1 presents the dates, students, discussants, and institutes involved.

**Table 1:** Students, discussants and institutes

Date	Name of the study	Presenter/institute	Discussant/institute
26/03/13	The feasibility of male involvement in prevention of mother to child transmission of HIV services in Blantyre, Malawi	Linda Nyondo-Mipando, Malawi University	Dr Simon Lewin, Norwegian Knowledge Centre for the Health Services, Norway
26/02/14	Analysis of association of SDH with reproductive health, focusing on contraceptive use and unplanned pregnancy among target couples in rural field practice area – RDGMC	Dr Shikha Sharma, RDGMC, UCTH, Ujjain	Dr Henry Lucas, Institute for Development Studies, UK
28/05/14	GIS (geographical information system) use in Health research system – an experience from MATIND	Prof Yogesh Sabde, RDGMC, UCTH, UJJAIN	Dr Merrick Zwarenstein, Western University/Karolinska Institutet, Sweden
28/08/14	Quality of obstetric referral services in India's JSY cash transfer programme for institutional births	Dr Sarika Chaturvedi, RDGMC, UCTH, UJJAIN and PhD Student, Global Health, Karolinska Institutet, Sweden	Dr Syed Abbas Institute for Development Studies, UK
			
27/10/14	GIS (Geographical Information System) use in Health research system – an experience from MATIND – Part 2	Prof Yogesh Sabde, RDGMC, UCTH, UJJAIN	Dr Merrick Zwarenstein, Western University/Karolinska Institutet, Sweden
19/08/15	Respectable, disreputable, or rightful? Young Nicaraguan women's discourses on femininity, intimate partner violence, and sexual abuse: a grounded theory situational analysis	Dr Mariano Salazar, Karolinska Institutet	Dr Ulla Ashorn, University of Tampere, Finland
11/11/15	Out-of-pocket expenditures for childbirth in the context of the Janani Suraksha Yojana (JSY) cash transfer program to promote facility births: Who pays and how much? Studies from Madhya Pradesh, India	Kristi Sidney, PhD Student, Global Health, Karolinska Institutet, Sweden	Dr Tetyana Stepurko, National University of Kyiv-Mohyla Academy, Sweden Dr Elizabeth Lutge, University of KwaZulu-Natal, South Africa
			

The research clinic sessions focused on a range of topics, within the wide themes of health systems and social determinants of health issues globally; examples were discussed from India to Malawi and Nicaragua. The students and researchers involved were doctoral students, postdocs, and junior staff. The presentations could be ongoing or completed work, or trials for papers intended for publication. A wide range of issues involving different experts was presented – for example, a student presented on out-of-pocket payments in India and received comments from experts from South Africa and Ukraine.

## Role of the student, commentator, and the audience

The students’ role was to send out their presentation; present their work; and respond to comments and queries from the commentator and the audience. The role of the commentator was key to the process: They carefully reviewed the students’ work and discussed their observations. They also invited questions from the audience and discussed the issue more generally. The audience numbers varied throughout the research clinics, and could range from five to approximately 20. The R D Gardi Medical College in India arranged research clinics so that students and faculty could join the meeting from one teleconferencing room; thus increasing attendance considerably when compared to single computer-point attendance. This could also offer benefits as the audience could discuss the research clinic after the meeting.

## Feedback from the students, commentators, and audience

According to student feedback, the students involved appreciated the opportunity to participate in research clinics and the comments from an international expert on their topic. According to some, the sessions boosted their confidence in presenting their work internationally, and provided additional mentoring, which is important for their career development (8). The format also allowed them to gain expertise internationally ‘face-to-face’, without the need for either to travel to meet. This is an important consideration given the environmental (9) and time impact of travelling. Students got useful and constructive feedback on their own work, through comments in the discussion, but often also in comments on their written work. Students and staff participating in the discussion on-site, or through web-links as audience also gained information and ideas from the discussion for their own work. They could get exposure to the kind of comments offered by international experts and reflect on their own work as students and researchers.

Though the consortium could see the benefit of the research clinic, organising them was not without challenges. The 16 partners across both ARCADEs were often in different time zones, university schedules differed, and other commitments of experts, students, and audiences meant that arranging a time to suit all was challenging. In addition, conducting these meetings via web-links in low- and middle-income contexts meant that bandwidth and other technological infrastructure impacted on both implementation and people's willingness to attend. Another key challenge was to get students the confidence to present their research and to instil trust in colleagues that their ideas will not be used by others. Though these issues were sometimes difficult and delayed the arrangement of the research clinics videoconferencing is widely available and in most settings bandwidth is improving. Conducting such sessions via the Internet is more practical than travelling for all concerned. The approach does need a dedicated person to take the project on, but this can easily be done alongside other research related tasks. With a general feeling of goodwill by researchers wanting to contribute to science, this method can support individual researchers and build networks.

## Conclusion

Despite the challenges to implementation, the research clinics concept succeeded in bringing together students and staff from different sides of the world. These connections can contribute toward research collaborations in the future, support young students and staff in low- and middle-income contexts, and thus support global capacity building in health research. It is hoped that mentees and participants involved will contribute to further capacity building in their home countries.

## Paper context

Low- and middle-income countries need research capacity to improve health systems. The African/Asian Capacity Development for Health Systems and Services Research/Research on Social Determinants of Health were large consortia that aimed to build the capacity of junior health researchers. This paper presents one way that researchers were mentored during the programme.
